# Arthroscopic Debridement and Lavage for Osteoarthritis of the Knee: Results From a Low-Resource Setting

**DOI:** 10.7759/cureus.31750

**Published:** 2022-11-21

**Authors:** Nachappa Sivanesan Uthraraj, Fitzgerald Anazor, Ali Hussain, Kumar Gaddam Raddy, Anand B Divekar, Raj Shrivastava, Jai Relwani

**Affiliations:** 1 Trauma and Orthopaedics, William Harvey Hospital, Ashford, GBR

**Keywords:** bmi, health care resources, knee, arthroscopic debridement, osteoarthritis

## Abstract

Introduction

Osteoarthritis of the knee is a highly prevalent disease globally, causing strain on healthcare resources and leading to a reduced quality of life. There are many treatments proposed for this condition, from conservative measures like analgesics and physiotherapy to surgical options like arthroscopy and total knee arthroplasty (TKA). Arthroscopic debridement and lavage provide significant improvement in a cohort of patients with particular features and can be a temporizing measure before TKA. This study aimed to investigate the results of this procedure, in a case series in the short-term and mid-term, in a low-resource setting.

Methods

This was a case series of 20 patients, who presented with clinical and radiographic features of mild to moderate (Kellgren-Lawrence grades I-III) primary osteoarthritis of the knee. Arthroscopic debridement and lavage were performed and the Knee Society Score (KSS) was recorded pre-operatively and post-operatively in the short and mid-term at one month, three months, and twelve months. Statistical analyses was done for correlation, with different variables such as the presence of meniscal pathology, loose bodies, grade of osteoarthritis, malalignment, and body mass index (BMI).

Results

The KSS improved at one month, three months, and twelve months for all the patients. The improvement in the KSS scores was associated with varus malalignment of less than 10 degrees, a BMI of less than 25, and the presence of loose bodies. There were no adverse events or complications from this study.

Conclusions

There was a significant improvement in a patient cohort with malalignment of less than 10 degrees, BMI of less than 25, meniscal pathology, and loose bodies. We can therefore recommend arthroscopic debridement and lavage as a temporizing measure before TKA in this particular cohort.

## Introduction

Osteoarthritis of the knee is a leading cause of disability worldwide; it reduces the quality of life and is associated with an increased incidence of depression. A study in Korea estimated that the number of years people live with osteoarthritis-associated disability is exceptionally high. Recent data from the United States demonstrated that 50% of people suffering from knee osteoarthritis are under 65 years of age, indicating this is no longer the geriatric condition it was once considered. Furthermore, it affects populations across the spectrum of socioeconomic backgrounds [1-4]. The etiological factors for osteoarthritis can be multifactorial but are predominantly related to articular cartilage wear secondary to injury or degeneration, which leads to further deterioration with movement, impact, and loading [5]. The most reliable and widely used classification for osteoarthritis of the knee is the Kellgren-Lawrence grading, based on radiographs of the knee joint [6]. Surgical options for osteoarthritis include debridement, lavage, chondroplasty, bone marrow stimulating techniques, chondrocyte transfer, chondrocyte implantation, and arthroplasty [7,8].

Arthroscopic debridement is an accessible treatment modality even in low-resource settings. It involves toilet lavage, chondroplasty, synovectomy, and meniscectomy and aims to improve symptoms by removing cartilaginous debris and inflammatory factors. It yields good results for mild to moderate osteoarthritis, particularly if the following factors are present: male gender, under 60 years old, symptomatic for fewer than six months, failure to respond to two months of conservative treatment, co-existing meniscal injury, minimal or no malalignment, or loose bodies. Previous studies have shown good to excellent outcomes in over 60% of individuals if the selection is based on these factors [9-12]. Its role as a temporizing procedure before total knee arthroplasty (TKA) is well documented [13-16]. However, its effectiveness has been questioned and there is no consensus on the outcomes of the procedure [17-19]. The Knee Society Score (KSS) is a reliable and validated tool, to evaluate the knee joint in terms of pain, range of motion, stability, and deformities [20]. This study aims to test the hypothesis that, arthroscopic debridement provides symptomatic relief and improvement of function in an appropriately selected cohort of mild to moderate osteoarthritic patients, by pre and post-operative KSS scores and that it has added value in the low-resource setting. 

## Materials and methods

We undertook a prospective cohort study of 20 patients in a low-resource, district general hospital in India. The Institutional Ethical Committee approved this study (JJMMC/4321/2022-23). Informed consent was obtained from all the participants in the study. The inclusion criteria were patients with osteoarthritic symptoms - demonstrated by clinical symptoms such as pain, stiffness, and mal-alignment; radiographic changes corresponding to Kellgren-Lawrence (KL) stages I-III, age from 40-80 years, failed conservative treatment of at least two months, and no previous surgical interventions for osteoarthritis of the knee. Weight-bearing radiographs of the symptomatic knee in antero-posterior and lateral views were taken. The clinical evaluation and the radiographic analysis were done by two independent orthopaedic surgeons. The exclusion criteria were any patient, who was already listed for a total knee replacement procedure, co-existence of any other rheumatological conditions, history of collateral or cruciate ligament deficiencies or surgery, and valgus angulation of more than 10 degrees. The surgical intervention was arthroscopic debridement and lavage without microfracturing. One antero-medial and one antero-lateral portal were used. The Stryker Arthroscopy Endoscopy camera system was used with the knee arthroscopy system from the same manufacturer (Stryker Corporation, Kalamazoo, MI, USA). The procedures were done by the same arthroscopy subspecialty-trained surgeon. The entire knee was inspected using the scope in the medial tibio-femoral, lateral tibio-femoral, and patello-femoral compartments including the medial gutter. The joint was debrided using the arthroscopic shaver. Loose bodies (if any) were extracted with the grasper tool. Partial meniscectomy and synovectomy were done as required, through the working portal. Lavage was done with a minimum of 6 liters of normal saline. None of the patients received any intra-articular steroid injections post-operatively. Nine patients (45%) had a degenerative meniscal tear which was discovered during arthroscopy. The patients were discharged on the same day of the operation (post-operative day 0) with no restrictions to weight bearing The KSS scores were obtained pre-operatively at short-term follow-up (one and three months), and at mid-term follow-up (12 months).

We did univariate and multivariate correlation analyses for improvement in the KSS post-operatively with the following variables - presence of loose bodies, degree of varus malalignment, grade of osteoarthritis, presence of meniscal pathology, and body mass index (BMI). We defined adverse events as peri-operative complications including bleeding, hemarthrosis, anaesthetic complications, surgical site infection, septic arthritis, deep vein thrombosis, and pulmonary embolism. The patients were discharged as day care surgical patients with an immediate follow-up appointment in one month. The statistical analysis was done using Microsoft Excel (Microsoft Corporation, Redmond, WA, USA) and IBM SPSS software, version 28 (IBM Corp., Armonk, NY, USA). 

## Results

The average age of our cohort was 65.4 years (S.D.=8.64) with 50% of the participants being male. Nine patients (45%) were found to have degenerative meniscal tears on arthroscopy (Table 1). Four patients had grade I osteoarthritis (20%), nine patients had grade II osteoarthritis (45%), and seven patients had grade III osteoarthritis (35%) (Table 2). The average BMI was 23.9 (S.D.=2.31). Six patients (30%) had loose bodies (Table 3). Fourteen patients had a varus angulation of <10 degrees, with the mean being 8.6 (S.D.=2.85). There were no adverse events or complications from this study. In performing the univariate analysis, the patients who had meniscal pathology experienced statistically significant improvements in the KSS score at one month (p-value <0.001), three months (p-value <0.001), and twelve months (p-value <0.001) (Table 4). There was also a significant correlation with grade III osteoarthritis at three and twelve months (p-value=0.018) and (p-value=0.017), respectively. The multivariate correlation analysis showed that the improvement in the KSS score at one month had a statistically significant correlation with varus angulation less than 10 degrees (p-value=0.03), at three months in patients with a BMI of less than 25 (p-value=0.05) and with loose bodies (p-value=0.046), and at twelve months in patients with loose bodies (p-value=0.015) and a BMI of less than 25 (p-value=0.042) (Table 5). None of the patients were dissatisfied with the results during the one-year follow-up and none were seeking other modalities of surgical intervention. 

**Table 1:** Meniscal pathology in the osteoarthritic knees

Meniscal pathology	Patients (%)
Present	9 (45%)
Absent	11 (55%)

**Table 2:** Kellgren-Lawrence grading of the osteoarthritic knees

Kellgren-Lawrence grading	Patients (%)
I	4 (20%)
II	9 (45%)
III	7 (35%)

**Table 3:** Presence of loose bodies in the osteoarthritic knees

Loose bodies	Patients (%)
Present	6 (30%)
Absent	14 (70%)

**Table 4:** Univariate correlation analysis of the post-operative KSS scores compared to the baseline KSS: Knee Society Score

Time after surgery	Variable	Beta value	Confidence interval	p-value
1 month				
	Meniscal pathology	8.8	4.4 to 13	<0.001
3 months				
	Meniscal pathology	11	6.6 to 15	<0.001
12 months				
	Meniscal pathology	13	7.7 to 18	<0.001

**Table 5:** Multivariate analysis of the correlation of improvement in the KSS score at different time periods compared to the baseline Knee Society Score

Time	Variable	Beta value	Confidence interval	p-value
1 month				
	Varus angulation <10 degrees	-4.5	-7.3 to -1.7	0.003
3 months				
	BMI <25	-0.86	-1.7 to 0.00	0.05
	Presence of loose bodies	-4.4	-8.8 to -0.09	0.046
12 months				
	Presence of loose bodies	-6.5	-12 to -1.4	0.015
	BMI <25	-1.1	-2.5 to -0.05	0.042

## Discussion

In our study, we were able to demonstrate that arthroscopic debridement yields significant positive results, when measured by the validated KSS, for patients with mild to moderate osteoarthritis (KL grades I-III), loose bodies, BMI of less than 25, less than 10 degrees of varus angulation and meniscal pathology. This was evident in the short and mid-term follow up. 

Our patients had consistent and significant improvement in their post-operative KSS scores over the short- and mid-term compared with their baseline. A systematic review and meta-analysis by Brignardello-Petersen compared arthroscopic debridement with conservative treatment or sham surgery in patients with symptomatic knees. It found moderate to high-quality evidence that arthroscopic debridement improved short-term (three months) pain, function, and quality of life [21]. A study performed in the Indian population yielded similar results; it found substantial short-term (two weeks, six weeks, and six months) and mid-term (12 months) improvement using the Visual Analog Scale (VAS), the International Knee Documentation Committee (IKDC) and the Short Form-8 (SF-8) tools [22]. A prospective cohort study by Lv et al. demonstrated improvements in resting pain and functional status for KL grades I-III with follow-ups up to two years using the VAS and the Hospital for Special Surgery Knee-Rating Scale (HSS) [23]. Our results of good functional and symptomatic improvement in patients with early-stage osteoarthritis (KL Grades I-III) are consistent with these results. 

We found a significant improvement in the results of patients who had meniscal lesions compared to those who did not. A prospective study by Giri et al. concluded that patients with meniscal and chondral lesions had better outcomes from arthroscopic debridement in the short- (six months) and mid-term (18 months) [24]. This was echoed in a prospective case series where Figueroa and colleagues recorded a good improvement in a series of 100 patients with meniscal pathology with a mean follow-up of 35.9 months. Of note, this study excluded patients with malalignment and used the Ahlback grading system (I-III) for inclusion [25]. 

The regular use of arthroscopic debridement for knee osteoarthritis decreased following the randomised control trial of 165 patients by Moseley et al, in 2002 [17]. Patients were randomised to one of three intervention groups: arthroscopic debridement, arthroscopic lavage, and placebo surgery. The study concluded that there was no significant difference between groups challenging the use of arthroscopic debridement and lavage for the treatment of knee osteoarthritis. However, methodological flaws leave the study’s conclusions open to scrutiny, as it was a single surgeon series, and assessments were based on the non-validated Specific Knee Pain Scale [17,26]. 

TKA is the established definitive treatment for osteoarthritis of the knee; however, it has certain disadvantages. A Study by Steadman et al. found that arthroscopic debridement was associated with less post-operative pain and shorter rehabilitation. TKA yielded better results in patients with more severe degenerative changes (KL III-IV). However, for patients with lesser grades of osteoarthritis, the risks of prosthetic joint infection and finite longevity of the implant likely outweigh the benefits of TKA [27]. A prospective case series of 1217 patients undergoing TKA by Scott et al. found that post-operative pain at the six-month follow-up resulted in patient dissatisfaction in roughly 20% of patients [13]. Our study demonstrates that arthroscopic debridement and lavage result in improved pain and function in select patients with early-stage osteoarthritis. It, therefore, has a role in symptom management and as a temporising measure before arthroplasty in this cohort. 

In the United States, the total annual average direct per patient treatment cost for knee osteoarthritis varied from USD 1442 to 21335; the skewed upper end of the range represents the costs involved in total knee replacement surgery [28]. A cost-benefit and Quality Adjusted Life Year analysis was significant for arthroscopic debridement with a cost under GBP 30000 [29]. These metrics are even more significant in a low-resource setting where services offered by the state are limited and there is no universal health insurance coverage. None of the patients in our series were seeking additional, costly, surgical management for their osteoarthritis, 12 months following arthroscopic debridement. In addition, the shorter recovery compared to TKA means younger patients can return to work sooner and incur less loss of earnings. The patients who underwent this intervention were discharged on the same day with instructions to weight bear, highlighting the benefits as a day-care surgery. This implies that in the short- to mid-term, arthroscopy was a cost-effective strategy for these patients. Furthermore, should the expense of a TKA be unavoidable, delaying the primary replacement may prevent the need for costly revision surgery. Our case series supports that arthroscopic debridement being offered before TKA reduces the economic burden on the patient. 

Patients who had a BMI of less than 25 fared well with arthroscopic debridement in our case series. Obesity has a role in accelerating the disease process of osteoarthritis [30]. There is no definitive data from other studies to benchmark a cut-off for good results with arthroscopic debridement to compare our results with. Based on our results, patients with early-stage arthritis and a normal BMI have improved outcomes from arthroscopic debridement and patient selection decisions should factor in BMI. 

There were no complications in terms of morbidity or mortality in our series. In a retrospective cohort study by Maletis et al., the reported incidence of complications including venous thromboembolism was 0.25% and pulmonary embolism was 0.17% in 20,770 patients who underwent elective knee arthroscopy. There was one surgically attributable death [31]. 

The strength of this study was that it was one of the very few studies to report this treatment for a low-resource Indian population and to look at the effect of BMI on the results. The drawbacks were that this is a single center series with only the KSS used as a tool to measure the outcomes.

## Conclusions

Knee osteoarthritis is a debilitating condition that can be a significant, if not life-changing, and economic burden to patients without National Health Service or universal healthcare. Our study supports existing evidence that for patients with specific existing knee pathologies and mild to moderate osteoarthritis, arthroscopic debridement and lavage is beneficial at least in the mid-term and as a temporizing procedure before TKA. Patient selection is key to improved patient outcomes and we report that normal BMI was significantly correlated with favourable outcomes. The results of our study promote the use of arthroscopic debridement as an accepted option in low-resource settings for cost-effectiveness.

In conclusion, we recommend arthroscopic debridement for the cohort of non-obese patients with early osteoarthritis of the knee, meniscal pathology, varus malalignment of less than 10 degrees, and loose bodies. This will provide significant improvements in pain and function. The long-term effects of this procedure will have to be investigated in a similar setting in the future. 
